# Thrombotic Paradox: Ischaemic Stroke in Immune Thrombocytopaenia. A Case Report and Review

**DOI:** 10.7759/cureus.1904

**Published:** 2017-12-03

**Authors:** Chong Yau Ong, Farhad F Vasanwala

**Affiliations:** 1 Family Medicine, Sengkang General Hospital, Sengkang Health, Singhealth, Singapore; 2 Department of General Medicine, Sengkang General Hospital, Sengkang Health, Singhealth, Singapore

**Keywords:** immune thrombocytopaenia, cerebral infarct, ischaemic stroke, idiopathic thrombocytopaenic purpura

## Abstract

Immune thrombocytopaenia (also known as idiopathic thrombocytopaenic purpura) (ITP) is a chronic condition with isolated low platelet counts. Although it is largely perceived that ITP predisposes to bleeding risks, thrombotic events, such as ischaemic strokes, do happen paradoxically in patients with ITP. A 68-year-old lady presented with right upper limb weakness and was diagnosed with an ischaemic stroke and was started on clopidogrel. She had a history of ITP. Two months later, she again had another ischaemic stroke. Prednisolone was added as her platelet count was below 50 x 10^9^/L. Based on this case and recent case studies, we suggest the administration of antiplatelet or anticoagulant agents judiciously if the platelet count is 50 x 10^9^/L or above with monitoring of bleeding risks. As for the management of ITP, we do agree with the general opinion that treatment is rarely required for patients with a platelet count above 50 x 10^9^/L. We recommend a haematology consult for discussion on treatment initiation if the platelet count is below 50 x 10^9^/L.

## Introduction

We present a case of recurrent ischaemic stroke in a middle-aged Singaporean woman with a background of immune thrombocytopaenia (ITP). To our knowledge, this is the first case reported of such in Southeast Asia; similar cases have been reported in America, United Kingdom, Australia, Japan, and Korea. Although the association between ischaemic stroke and immune thrombocytopaenia is becoming increasingly known, this is still a rare condition, given the paucity of number cases reported globally.

The management of ischemic stroke in the setting of ITP remains a challenge, despite the increased clarity and insight on the pathogenesis of paradoxical thrombosis. There is no consensus on how ischemic stroke on ITP should be managed. Nevertheless, the authors believe that the patients should be managed judiciously, balancing between treating the thrombotic event and monitoring for bleeding risks.

## Case presentation

A 68-year-old woman presented with increased right upper limb weakness for one week's duration. There were no sensory symptoms. Her vital signs were normal. Examination showed slurring of speech with decreased power in the right upper limb proximal muscle strength (Medical Research Council (MRC) score of 4) compared to the left (MRC 5). Muscle power in the distal upper limbs was equal. Power was also decreased in the right proximal lower limb compared to the left. The patient had a past medical history of old ischaemic stroke in 2006, hypertension, dyslipidaemia, Graves’ disease, and immune thrombocytopaenia (ITP), which was diagnosed incidentally in 2008. There was no bleeding manifestation of the ITP on diagnosis; her platelet was repeatedly below 100 x 10^9^/L at that time. There were no immature or abnormal white cells, no schistocytes, and no abnormal platelet morphology on blood film. Coagulation tests were not prolonged. Antiphospholipid antibodies (lupus anticoagulant and anticardiolipin), anti-double-stranded DNA, and anti-Smith antibodies were negative. Complements (C3 and C4) were normal. Human immunodeficiency virus (HIV) and hepatitis C antibodies were not detected. Helicobacter pylori screening was not done.

**Figure 1:**
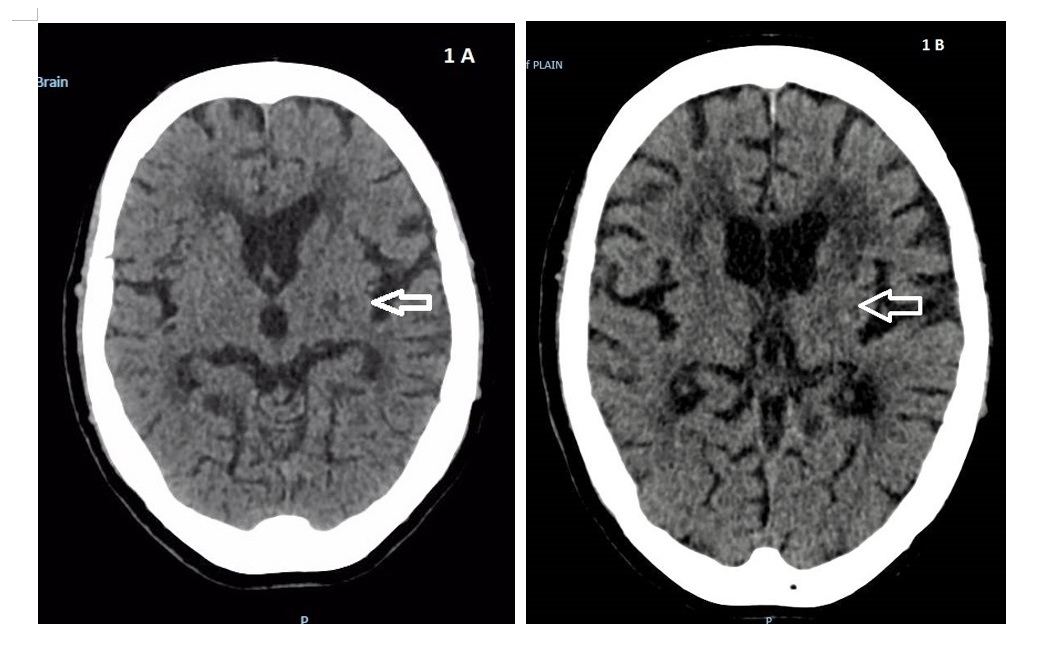
Computed Tomography (CT) of the Brain 1A (left): CT brain on first presentation showing hypodensity in the left corona radiata. 1B (right): CT brain near three months later showing hyperdensity in the left corona

Computed tomography (CT) of the brain showed hypodensity in the left corona radiata and old infarcts with a background of small vessel disease (Figure 1A). A decision was made by the neurologists not to proceed with magnetic resonance imaging (MRI) of the brain in view of the multiple old strokes. A diagnosis of right ataxic hemiparesis was made. An ultrasound of her carotid arteries showed moderate left and mild right carotid artery stenosis. Platelet level at the onset of stroke was 119 x 10^9^/L (reference range 150-400 x 10^9^/L). Total white cells were 6.98 x 10^9^/L and haemoglobin 14.4 g/dL. Thrombophilia screenings revealed levels of Factor V Leiden, proteins C and S, and antithrombin were not deficient. She was not on prednisolone. After consultation with a haematologist, the decision was made to start clopidogrel. She underwent a one-month duration of rehabilitation. While on clopidogrel, her platelet count dropped to a nadir of 59 x 10^9^/L. There were no clinical symptoms or signs of bleeding manifested. Antiplatelet therapy was continued. She was discharged ambulant with a walking frame.

Two months later, she presented with acute right-sided lower limb weakness and difficulty in walking, which was new to her. CT brain did not show any acute haemorrhage. Multiple chronic lacunar infarcts were seen in bilateral thalami, internal capsules, corona radiata, and pons. MRI with diffusion-weighted imaging (DWI) did not show the presence of an acute infarct. There were multiple chronic infarcts. Multiple microhaemorrhages were also seen. Magnetic resonance angiography (MRA) showed diffuse flow irregularities in the anterior and posterior circulations due to atherosclerosis without significant flow-limiting stenosis. She was diagnosed with right ataxic hemiparesis and DWI-negative stroke with microhaemorrhages. Her platelet count was 41 x 10^9^/L, and the clopidogrel was suspended. She was started on prednisolone, 60 mg daily, as advised by the haematologist. Subsequent platelet counts were 43 x 10^9^/L and 51 x 10^9^/L before the clopidogrel was restarted with esomeprazole. Her platelet remained stable at 88 to 126 x 10^9^/L during the course of rehabilitation, and her prednisolone was gradually tapered to 20 mg daily upon discharge.

Three weeks after discharge from above-mentioned admission, she was admitted for non-specific breathlessness. Initial investigations showed that her blood gases and chest x-ray were normal. A CT scan was performed because the patient complained of right-sided weakness with deconditioning of her activities of daily living, to the point where she needed a wheelchair for ambulation. CT showed no acute intracranial haemorrhage or territorial infarct. A 0.4 cm hyperdense lesion was noted in the left corona radiata that corresponded to a prior punctate haemorrhage (Figure 1B). Her platelet count was 127 x 10^9^/L. She was discharged uneventfully with clopidogrel and same previous prednisolone dose of 15 mg daily.

Literature review

We reviewed recently published reports on adult ischaemic stroke in ITP over the past five years (from 2012 onwards) using PubMed search and hand search with Google. Keywords of ischaemic stroke and ITP were used. There were seven reports published [1-7], and we included our report in the table summarised below (Table 1).

**Table 1:** Summary of Published Case Reports of Ischaemic Stroke in ITP in the Last Five Years ITP: immune thrombocytopaenia; F: female; M: male

Author	Demographic (age/sex)	Clinical presentation	Platelet count on stroke onset (10^9^/L)	Treatment of ITP after stroke	Treatment of stroke
Our patient	68/ F	Right upper limb weakness	119	Prednisolone	Clopidogrel
Yunoki, et al. [1]	31/ F	Disorientation	132	Not given	Aspirin
Ichijo, et al. [2]	60/ F	Right-sided weakness, right hemispatial neglect	20	Prednisolone, dexamethasone, cyclosporine	Cilostazol
Kim H, et al. [3]	46/ F	Left-sided weakness	20	Dexamethasone	Not given
De La Pena, et al. [4]	84/ M	Aphasia, dysarthria	40	Prednisolone	Not given
Choi, et al. [5]	58/ F	Vertigo, gait disturbance	347	Data unavailable	Aspirin
Tsuda, et al. [6]	41/ F	Vertigo, gait disturbance	71	Data unavailable	Sodium ozagrel
Zhao, et al. [7]	36/ M	Right-sided weakness and numbness (transient), vertigo	6	Thrombopoietin	Not given

## Discussion

In 1735, Werlhof first described a syndrome similar to autoimmune thrombocytopaenia in a young woman with cutaneous petechiae, ecchymoses, and haemorrhages in mucous membranes before the discovery of platelets. Immune thrombocytopaenia (also known as idiopathic thrombocytopaenic purpura) is a chronic condition whereby the platelet count is below 100 x10 ^9^/litre without concomitant anaemia or leucopaenia. The American Society of Haematology defined ITP as isolated thrombocytopenia with no clinically apparent associated conditions or other causes of thrombocytopenia, e.g., human immunodeficiency virus infection, systemic lupus erythematosus, lymphoproliferative disorders, myelodysplasia, and agammaglobulinaemia [8]. Therefore, the diagnosis is clinical and based on a diagnosis of exclusion of the above medical conditions. The estimated incidence of ITP in American adults is about 3.3 new cases per 10^5^ adults/year.

Stroke is the initial presentation of 1.27% of patients with haematological disorders, in particular, disorders with a prothrombotic state (essential thrombocytopaenia, primary and secondary polycythaemia, myeloma, and leukaemia). This is likely so with our patient who had her first stroke preceding the diagnosis of ITP, although the causality is also compounded by cardiovascular risk factors for dyslipidaemia and hypertension.

Platelet microparticles (PMPs) are the best model currently available to explain the mechanism of thrombosis in ITP patients. PMPs are released by the platelets upon activation and the level represents biologic markers in patients with acute ischaemic stroke. Elevated levels of PMPs are detected in patients with ITP and concurrent transient ischemic attacks (TIAs) or ischemic strokes, as compared to normal healthy controls [9]. Microparticles are also recognised as a major role player in the pathogenesis of coronary artery disease. Microparticles are the main mediators of inflammation; they promote endothelial intracellular adhesion molecule 1 (ICAM-1)-dependent monocyte adhesion and transendothelial migration of plaques. Both formation and progression of atherosclerosis and plaque rupture can be attributed to the actions of microparticles.

Other contributory markers identified are circulating platelet-leukocyte-monocyte aggregates, endothelium-activating antibodies, the activation of the complement system, and low levels of disintegrin and metalloproteinase with thrombospondin type 1 motif, member 13 (ADAMTS-13). The association with other diseases, such as antiphospholipid syndrome (APS), also puts patients with ITP at significant thrombotic risk. The presence of a lupus anticoagulant in the absence of APS is known to increase the risk of thrombosis among patients with ITP.

Another arm of thrombosis predisposition is the treatment of ITP itself. Intravenous immunoglobulin (IVIG) is associated with acute ischaemic stroke, in which cases of acute ischaemic stroke have been reported to happen as early as four hours, peaking in the first 24 hours of infusion, and lasting up to seven days after treatment. The possible mechanism is postulated to be due to increased viscosity of plasma after IVIG administration and IVIG-induced vasospasm. Treatment with corticosteroids, which is commonly used in ITP, may also play role in tipping the patient into a hypercoagulable state. Thrombotic events have been also observed in patients post-splenectomy, a second-line treatment for ITP.

Managing ischaemic stroke with underlying ITP remains an area of contention. Review of recently published case reports of ischaemic strokes in ITP showed that antiplatelet therapy was initiated in more than half of the cases. However, no concrete consensus can be drawn based on the limited number of patients studied. The treatment modality of acute ischaemic stroke with antiplatelet and anticoagulant agents may risk concurrent and imminent thrombocytopaenia. Bleeding tendencies with a platelet count of less than 100,000/mm^3^ are an absolute contraindication for thrombolysis with tissue plasminogen activator in acute ischaemic stroke. Also, the use of antiplatelet and anticoagulant agents has not been proven to have significant effects on levels of PMPs. For the management of ITP per se, The American Society of Haematology suggests treatment be initiated for newly diagnosed patients with platelet counts less than 30 x 10^9^/L [10].

## Conclusions

Despite the availability of stroke pathways and recommendations for ischaemic stroke, the guidelines have been silent on managing acute ischaemic stroke in patients with underlying ITP. The need for further systematic research in comparing the acute, intermediate, and long-term effects of current treatment modalities is clearly evident.

We suggest the administration of antiplatelet or anticoagulant agents judiciously if the platelet count is 50 x 10^9^/L or above with monitoring of bleeding risks. As for the management of ITP, we do agree with the general opinion that ITP-specific treatment is rarely required for patients with platelet counts above 50 x 10^9^/L. We recommend a haematology consult for discussion on treatment initiation if the platelet count is below 50 x 10^9^/L. For general and practical application when dealing with decisions pertinent to management, the cardinal methods of weighing the patient’s risk factors, lifestyle, the risk of bleeding transformation, and noting the patient's individual preference should be adopted.
